# Fibre intake and the development of inflammatory bowel disease: A European prospective multi-centre cohort study (EPIC-IBD)

**DOI:** 10.1093/ecco-jcc/jjx136

**Published:** 2017-10-07

**Authors:** Vibeke Andersen, Simon Chan, Robert Luben, Kay-Tee Khaw, Anja Olsen, Anne Tjonneland, R Kaaks, Olof Grip, M M Bergmann, H Boeing, Johan Hultdin, Pontus Karling, Kim Overvad, Bas Oldenburg, Jorrit Opstelten, Marie-Christine Boutron-Ruault, Franck Carbonnel, Antoine Racine, Timothy Key, Giovanna Masala, Domenico Palli, R Tumino, A Trichopoulou, Elio Riboli, Andrew Hart

**Affiliations:** 1 Laboratory Center, Hospital of Southern Jutland, Denmark; 2 Institute of Regional Health Research - Center Sønderjylland, University of Southern Denmark, Denmark; 3 Institute of Molecular Medicine, University of Southern Denmark, Denmark; 4 Department of Medicine, Norwich Medical School University of East Anglia, UK; 5 Norfolk & Norwich University Hospitals NHS Trust, UK; 6 Strangeways Research Laboratory, Institute of Public Health, University of Cambridge, UK; 7 Danish Cancer Society Research Center, Danish Cancer Society, Denmark; 8 Division of Clinical Epidemiology, DKFZ - German Cancer Research Centre, Germany; 9 Department of Clinical Sciences, University Hospital, Sweden; 10 Department of Epidemiology, German Institute of Human Nutrition, Germany; 11 Department of Medical Biosciences, Clinical Chemistry, Umeå University, Sweden; 12 Department of Public Health and Clinical Medicine, Umeå University, Sweden; 13 Department of Public Health, Section for Epidemiology, Aarhus University, Denmark; 14 Department of Gastroenterology and Hepatology, University Medical Center Utrecht, The Netherlands; 15 INSERM, Centre for Research in Epidemiology and Population Health, Institut Gustave Roussy, France; 16 Universite Paris Sud, UMRS 1018, France; 17 Department of Gastroenterology, Bicetre University Hospital, Assistance Publique des Hopitaux, France; 18 Cancer Epidemiology Unit, Nuffield Department of Population Health, University of Oxford, UK; 19 Cancer Risk Factors and Life-Style Epidemiology Unit, Cancer Research and Prevention Institute – ISPO, Italy; 20 Cancer Registry and Histopathology Department, ‘Civic - M.P.Arezzo’ Hospital, Italy; 21 WHO Collaborating Centre for Food and Nutrition Polices, Greece; 22 Division of Epidemiology, Imperial College London, UK

**Keywords:** Dietary fibre, diet, epidemiology, fibre food, inflammatory bowel disease, prospective study

## Abstract

**Background and Aims:**

Population-based prospective cohort studies investigating fibre intake and development of inflammatory bowel disease are lacking. Our aim was to investigate the association between fibre intake and the development of Crohn’s disease [CD] and ulcerative colitis [UC] in a large European population.

**Methods:**

In total, 401326 participants, aged 20–80 years, were recruited in eight countries in Europe between 1991 and 1998. At baseline, fibre intake [total fibres, fibres from fruit, vegetables and cereals] was recorded using food frequency questionnaires. The cohort was monitored for the development of inflammatory bowel disease. Each case was matched with four controls and odds ratios [ORs] for the exposures were calculated using conditional logistic regression. Sensitivity analyses according to smoking status were computed.

**Results:**

In total, 104 and 221 participants developed incident CD and UC, respectively. For both CD and UC, there were no statistically significant associations with either quartiles, or trends across quartiles, for total fibre or any of the individual sources. The associations were not affected by adjusting for smoking and energy intake. Stratification according to smoking status showed null findings apart from an inverse association with cereal fibre and CD in non-smokers [Quartile 4 vs 1 OR = 0.12, 95% confidence interval = 0.02–0.75, *p* = 0.023, OR trend across quartiles = 0.50, 95% confidence interval = 0.29–0.86, *p* = 0.017].

**Conclusion:**

The results do not support the hypothesis that dietary fibre is involved in the aetiology of UC, although future work should investigate whether there may be a protective effect of specific types of fibre according to smoking status in CD.

## 1. Introduction

The inflammatory bowel diseases [IBDs] Crohn’s disease [CD] and ulcerative colitis [UC] are both chronic inflammatory diseases of the gastrointestinal tract with relapsing courses. They have a large impact on patients, their families and society due to absences from work and healthcare expenses. The incidence of IBD is rising in both Western and developing countries,^1^ so preventive strategies based on knowledge of their aetiology are required. The increasing incidence suggests a role for environmental exposures acting either alone or by interacting with genetic factors. Candidate gene studies and genome-wide investigations have identified more than 230 loci involved in IBD including *NOD2* and *IL10*,^2^ and epidemiological studies have reported associations with environmental risk factors such as smoking, hygiene, antibiotics and diet.^3^ Currently, there considerable interest in the impact of diet on IBD.^4–6^

For dietary fibre, there are several plausible biological mechanisms for how this may prevent the development of IBD. Firstly, dietary fibre found in fruit, vegetables, cereals and legumes has a bulk function leading to reduced intestinal transit times, so potential toxic exposures have less time in contact with the intestinal wall. Secondly, fibre is converted by colonic bacteria to short-chain fatty acids [SCFAs], including butyrate, which may ameliorate intestinal inflammation.^7,8^ Thirdly, dietary fibre may influence the composition of the intestinal microbiota,^9^ which is altered in patients with IBD,^10^ and fourthly soluble plant fibres are involved in maintaining the intestinal barrier function.^11^ These potential protective mechanisms need to be supported by epidemiological studies showing that initially healthy individuals with a high dietary fibre intake subsequently have a low risk of developing IBD. The preferred methodology is a cohort study, which reduces both selection and recall biases that are inherent in case-control investigations. To date, there is only one such investigation, a large prospective study in 170776 women in the US Nurses’ Health Studies [NHS].^12^ The highest quintile of fibre intake [median 24.3 g/day] was associated with a 41% lower risk of CD compared to the lowest quintile.^12^ The risk was lowest for fibres derived from fruits, whereas fibres from vegetables, cereals, whole grains or legumes did not modify risks.^12^ For UC, there were no associations with either total or specific types of fibres.^12^ Retrospective studies of fibre and disease onset, and clinical relapse of IBD did not find any consistent effects.^5,13,14^ The inconsistencies regarding the role of fibre in the current literature need to be clarified in further population-based prospective cohort studies.

The aim of this investigation was to study if there was an inverse association between dietary fibre intake and the development of CD and UC, for the first time in a population of both men and women in a large prospective cohort study. Furthermore, we evaluated the association for fibres from several sources to suggest any potential mechanisms through which different fibres may reduce IBD risk and whether there was effect modification by smoking, the most consistently documented risk factor for IBD.^15^

## 2. Material and methods

The ‘EPIC-IBD’ Study cohort [European Prospective Investigation into Cancer and Nutrition-IBD study] is embedded in the main EPIC study, which consists of 521000 men and women aged 20–80 years, who were recruited from 23 European centres in ten western European countries.^16^ EPIC was designed to study the relationship between diet and cancer and other chronic diseases. The design and methods of the EPIC project have been described elsewhere in detail.^17,18^ The EPIC-IBD study cohort is a sub-cohort of 401326 participants without IBD at enrolment, recruited from eight countries [Denmark, France, Germany, Greece, Italy, the Netherlands, Sweden and UK], enrolled between 1991 and 1998^19–21^ [Appendix 1]. At baseline, participants completed detailed questionnaires which provided information on their age, gender, smoking and physical activity over the previous 12 months before recruitment. Participants also completed country-specific validated food frequency questionnaires [FFQs], which contained a list of approximately 200 foods and nine frequency of intake categories varying from *never* eating to *several times per day*. Recordings of foods were converted to nutrient intake using the European Nutrient Database, a method for estimating total dietary fibre intake.^22^ Intakes of total daily dietary fibre and fibre from fruits, vegetables and cereals were calculated. Data on total daily energy intake were also derived from the FFQ. To ensure compatibility between the FFQs used in each centre, a calibration study was performed using a computer-assisted 24-h dietary recall in a sub-sample of each population in each centre on stratified random samples of 36900 subjects.^18,23^ This was used to correct for any systematic over- or under-estimates of the baseline food frequency questionnaires used and ensure the data could be combined.

The cohort was monitored after recruitment until at least June 2004, and in some centres until 2010, to identify those participants who developed incident CD and UC by linkages with either national or regional registries, follow-up questionnaires. and hospital pathological and radiological databases. All potential cases were reviewed by clinicians to confirm the diagnoses and disease extent within the gastrointestinal tract from the medical records.^21^ Cases of indeterminate or microscopic colitis were excluded, as were those with prevalent IBD at recruitment and participants diagnosed with IBD < 18 months after recruitment. The latter helped to ensure the dietary data reflected that prior to the development of symptoms, and for a period between the onset of symptoms and diagnosis.^21^ Each case was matched with four randomly selected controls matched for gender, age at recruitment [± 6 months], centre and recruitment date [±3 months]. Controls also had to be alive on the date of diagnosis of their matched case to ensure similar follow-up times. None of the controls at recruitment had either CD, UC, or microscopic or indeterminate colitis.

In the descriptive analyses, continuous variables were described with a mean and standard deviation, or median and range, according to the nature of the distributions. Categorical variables were presented as percentages. Differences between cases and controls were compared with either a Student’s *t*-test, Mann–Whitney test or chi-square^2^ test. The intakes of dietary fibres were divided into quartiles across the distribution of the whole cohort, with the lowest intake as the reference. The analytical method was a nested case–control one within a cohort study.^17^ The odd ratios [ORs] with 95% confidence intervals [CIs] were calculated using conditional logistic regression [STATA version 11] for intake of fibre and the development of either CD or UC, with and without adjusting for smoking and total energy intake, both measured at recruitment. A sensitivity analysis was performed for fibre intakes according to the most consistently documented risk factor for IBD, namely smoking status^15^ [firstly in non-smokers and secondly in the combined categories of former smokers and current smokers].

The research protocols were approved by local ethics committees and subjects gave written informed consent for access to their medical data.

## 3. Results

### 3.1. Crohn’s disease

In total, 104 participants developed incident CD [73.1% women, mean age at diagnosis = 55.1 years, SD = 11.1 years]. The median time between recruitment and diagnosis was 5.3 years [range 1.5–12.3 years] [Table 1]. In most cases [90.4%] the disease location was recorded, with similar proportions in either the ileum, colon or ileocolonic regions. Cases were more likely than controls to be a current smoker. The FFQs recording diet were complete for all types of fibre for 99% of cases and 99% for controls. There were no differences in the median intakes of either total or the specific types of fibres between cases and controls. In the analyses, there were no associations with either any quartiles, or trends across quartiles, for total fibre intake or for any of the three sub-types and the odds of developing CD [Table 2]. Adjusting for smoking and energy intake did not alter the magnitude of most associations, and had no effect on statistical significance. In sensitivity analyses stratified according to smoking status, in the combined group of former or current smokers [*n* = 66, 63.5% cases] there were no associations with any quartile of fibre intakes or trends across quartiles [data not shown]. For never smokers [*n* = 38, 36.5% of cases] there were similarly no associations apart for fibre from cereals, where an inverse association was observed for the two highest quartiles of fibre intakes above the reference with the odds of developing CD [Q3 vs Q1 OR = 0.18, 95% CI = 0.26–1.27, *p* = 0.086 and Q4 vs Q1 OR = 0.12, 95% CI = 0.02–0.75, *p* = 0.023], and a significant trend across quartiles (hazard ratio [HR] trend = 0.50, 95% CI = 0.29–0.86, *p* = 0.017) [Table 3].

**Table 1.** Baseline characteristics of Crohn’s disease [CD] and controls

	CD cases [*n* = 104]	Controls [*n* = 416]
Female [*n*, %]	76 [73.1]	304 [73.1]
Age [years] at recruitment [mean, SD]	49.6 [10.7]	49.6 [10.6]
Age [years] at diagnosis [mean, SD]	55.1 [11.1]	–
Time to diagnosis [years] [mean, range]	5.3 [1.5–12.3]	
Distribution of disease [*n*, %]		
L1, ileal	30 [31.9]	–
L2, colonic	35 [37.2]	–
L3, ileocolonic	26 [27.7]	–
L4, isolated upper GI disease	3 [3.2]	–
[unknown]	[*n* = 10]	–
Smoking status [*n*, %]		
never smoked	38 [36.5]	197 [47.4]*
past smoker	28 [26.9]	117 [28.2]
current smoker	38 [36.5]	102 [24.5]*
Dietary intakes [median, range]		
Total energy [kcal/day]	2117.4 [789.6–4312.4]	2071.7 [900.1–4795.4]
Total fibre [g/day]	22.3 [10.0–40.3]	21.9 [9.2–44.6]
Fruit fibre [g/day]	3.1 [0.2–12.9]	3.4 [0.3–16.7]
Vegetable fibre [g/day]	3.7 [0.5–13.1]	3.8 [0.4–16.9]
Cereal fibre [g/day]	7.5 [1.7–20.0]	7.8 [1.2 – 25.5]

*p < 0.001. There were no significant differences in any fibre intakes between cases and controls.

**Table 2.** Fibre intakes and the odds of developing Crohn’s disease

Fibre type [g/day, range]	Controls [*n*]	Cases [*n*]	OR [95% CI]	OR [95% CI]*
Total fibre				
7.1–<17.3	104	26	1.00	1.00
17.3–<21.7	108	21	0.78 [0.41–1.47]	0.78 [0.40–1.51]
21.7–<26.4	98	32	1.33 [0.73–2.42]	1.49 [0.75–2.94]
26.4–57.6	104	25	0.98 [0.53–1.82]	0.83 [0.38–1.81]
Fruit fibre				
0.1–<1.9	101	28	1.00	1.00
1.9–<3.4	102	27	0.93 [0.50–1.74]	0.96 [0.51–1.82]
3.4–<5.7	107	22	0.74 [0.39–1.39]	0.76 [0.40–1.46]
5.7–23.4	102	26	0.90 [0.45–1.81]	0.85 [0.40–1.79]
Vegetable fibre				
0.1–<2.3	105	25	1.00	1.00
2.3–< 3.7	101	28	1.15 [0.60–2.18]	1.08 [0.56–2.08]
3.7–<5.5	102	27	1.09 [0.55–2.17]	1.03 [0.50–2.10]
5.5–22.8	106	23	0.84 [0.38–1.84]	0.76 [0.33–1.74]
Cereal fibre				
0.9–<5.4	103	27	1.00	1.00
5.4–<7.8	100	29	1.08 [0.59–1.97]	1.10 [0.59–2.03]
7.8–<11.3	110	20	0.67 [0.34 -1.34]	0.65 [0.32–1.35]
11.3–28.0	101	28	1.04 [0.52–2.07]	0.99 [0.47–2.09]

*Adjusted for smoking status and energy intake. OR, odds ratio; CI, confidence interval.

**Table 3.** Fibre intakes and the odds of developing Crohn’s disease according to smoking status

Fibre type [g/day, range]	Non-smokers cases [*n*]	Non-smokers OR [95% CI]	Former or current smokers cases [*n*]	Former or current smokers OR [95% CI]
Total fibre				
7.1–<17.3	9	1.00	17	1.00
17.3–<21.7	4	0.37 [0.07–1.87]	17	0.87 [0.36–2.10]
21.7–<26.4	14	0.61 [0.19–2.01]	18	1.72 [0.59–4.99]
26.4–57.6	11	0.22 [0.04–1.09]	14	1.07 [0.36–3.23]
Fruit fibre				
0.1–<1.9	7	1.00	21	1.00
1.9–<3.4	12	1.07 [0.24–4.77]	15	0.99 [0.41–2.39]
3.4–<5.7	7	0.73 [0.13–3.98]	15	0.99 [0.42–2.35]
5.7–23.4	12	0.68 [0.15–3.09]	14	0.84 [0.26–2.70]
Vegetable fibre				
0.1–<2.3	9	1.00	16	1.00
2.3–< 3.7	7	0.39 [0.07–2.11]	21	1.66 [0.70–3.92]
3.7–<5.5	11	0.38 [0.10–1.49]	16	1.30 [0.46–3.65]
5.5–22.8	10	0.34 [0.08–1.41]	13	0.84 [0.21–3.39]
Cereal fibre				
0.9–<5.4	9	1.00	18	1.00
5.4–<7.8	16	1.67 [0.52- 5.39]	13	0.43 [0.14–1.33]
7.8–<11.3	5	0.18 [0.26 -1.27]	15	0.52 [0.18–1.53]
11.3–28.0	8	0.12 [0.02- 0.75]*	20	1.13 [0.39–3.32]

The odds ratios [ORs] are adjusted for total energy intake. CI, confidence interval.

*Trend across quartiles OR = 0.50, 95% CI = 0.29–0.89, *p* = 0.17.

### 3.2. Ulcerative colitis

A total of 221 participants developed incident UC [58.4% women, mean age at diagnosis = 57.9 years, SD = 10.4 years]. The median time between recruitment and diagnosis was 4.8 years [range 1.7–15.2 years] [Table 4]. Approximately two-thirds of participants had disease up to the splenic flexure. Cases were more likely than controls to be past smokers and current smokers. The FFQs recording diet were complete for all types of fibre for 98% of cases and for 99% of controls. There were no differences in the median intakes of either total or the specific types of fibres between cases and controls. In the analyses, there were no associations with either quartiles or trends across quartiles for total fibre intake or that for any of the three sub-types and the odds of developing UC [Table 5]. Adjusting for smoking and energy intake did not alter the magnitude of most associations, and had no effect on statistical significance. In sensitivity analyses stratified according to smoking status, there were no associations with any quartiles of fibre intake or trends across quartiles [data not shown].

**Table 4.** Baseline characteristics of ulcerative colitis [UC] cases and controls

	UC cases [*n* = 221]	Controls [*n* = 884]
Female [*n*, %]	129 [58.4]	521 [58.9]
Age [years] at recruitment [mean, SD]	51.6 [10.5]	51.8 [10.5]
Age [years] at diagnosis [mean, SD]	57.9 [10.4]	–
Time to diagnosis [years] [mean, range]	4.8 [1.7–15.2]	
Distribution of disease [*n*, %]		
E1, ulcerative proctitis	51 [27.0]	–
E2, left sided colitis	79 [41.8]	–
E3, extensive colitis	59 [31.2]	–
[extent not determined]	[*n* = 32]	–
Smoking status [*n*, %]		
never smoked	59 [26.7]	388 [43.9]*
past smoker	85 [38.5]	262 [29.6]**
current smoker	77 [34.8]	234 [26.5]**
Dietary intakes [median, range]		
Total energy [kcal/day]	2132.5 [982.0–4060.0]	2065.5 [955.9–4333.7]
Total fibre [g/day]	22.0 [9.2–42.4]	21.9 [8.2–51.6]
Fruit fibre [g/day]	3.8 [0.3–16.8]	3.5 [0.3–14.3]
Vegetable fibre [g/day]	3.7 [0.4–12.7]	3.8 [0.5–13.4]
Cereal fibre [g/day]	8.5 [1.5–24.7]	8.1 [1.6–25.4]

*p < 0.001, **p = 0.01. There were no significant differences in any fibre intakes between cases and controls.

**Table 5.** Fibre intakes and the odds of developing ulcerative colitis

Fibre type [g/day, range]	Controls [*n*]	Cases [*n*]	OR [95% CI]	OR [95% CI]*
Total fibre				
6.4–<17.3	228	47	1.00	1.00
17.3–<21.9	214	61	1.36 [0.90–2.08]	1.39 [0.88–2.19]
21.9–<27.1	221	54	1.21 [0.78–1.88]	1.20 [0.73–1.99]
27.1–75.2	220	55	1.21 [0.78–1.88]	1.22 [0.71–2.08]
Fruit fibre				
0.1–<2.2	223	52	1.00	1.00
2.2–<3.6	224	51	0.98 [0.64–1.51]	1.02 [0.65–1.58
3.6–<5.9	214	61	1.24 [0.81–1.91]	1.27 [0.82–2.00]
5.9–49.2	221	53	1.03 [0.65–1.63]	1.08 [0.67–1.77]
Vegetable fibre				
0.3–<2.5	216	59	1.00	1.00
2.5–<3.9	224	51	0.82 [0.54–1.28]	0.80 [0.51–1.25]
3.9–<5.8	217	58	0.97 [0.63–1.52]	0.96 [0.61–1.50]
5.8–25.7	226	49	0.78 [0.48 -1.28]	0.70 [0.41–1.18]
Cereal fibre				
0.1–<5.6	225	50	1.00	1.00
5.6–<8.1	220	55	1.14 [0.73–1.77]	1.19 [0.75–1.88]
8.1–<11.5	217	58	1.20 [0.77–1.87]	1.22 [0.76–1.97]
11.5–40.7	221	54	1.10 [0.68–1.78]	1.09 [0.64–1.986]

*Adjusted for smoking status and energy intake. OR, odds ratio; CI, confidence interval.

## 4. Discussion

The main finding of this study was that there were no associations in the whole cohort with the intakes of total fibre, or fibre from fruit, vegetables or cereals, and the subsequent development of either CD or UC.

These null findings are despite the potential plausible biological mechanisms, including fibre decreasing intestinal transit times and thereby reducing the time for contact of the gastrointestinal mucosa with potential causative agents, the anti-inflammatory effects of fibre both systemically and locally including in animal models reporting dietary fibre reducing gut inflammation,^8^ and potential effects on the microbiome and intestinal barrier. In the large bowel, there is microbial degradation of fibre to SCFAs, which have many anti-inflammatory actions in the gut including regulation of leukocyte functions such as migration and production of cytokines.^7^ Dietary fibre has been reported to stimulate the production of the anti-inflammatory interleukin 10 [IL-10]^7,8^ and a genetic marker has been identified near *IL10* as a risk marker for CD and UC.^2,24^ Interestingly, interactions between fibre intake and this genetic locus have also been found in relation to colorectal cancer.^25,26^ Although we documented no associations with fibre in the whole cohort we suggest future work should investigate whether diet–gene interactions may have a role in IBD, similar to colorectal cancer.^26^ Certainly, in paediatric CD, the risk associated with a high ratio of dietary intake of *n*-6 to *n*-3 polyunsaturated fatty acids occurs only in those who are carriers of specific variants of genes that control the metabolism of these macronutrients, namely *CYP4F3* and *FADS2*.^27^ Whether fibre has an effect in subgroups of people with a particular genetic profile, possibly related to *IL10*, is unknown. Furthermore, dietary fibre may influence the intestinal microbiome, which is probably important in the aetiology of IBD with documented increases in Enterobacteriaceae and reduced proportions of Firmicutes and Bacteroides.^10^ Differences in fibre intake have been postulated to explain variations in the intestinal microbiota between children from Europe and Africa.^9^ Also, dietary plant fibre inhibits the translocation of CD-associated bacteria across cultured intestinal Caco2 cells.^11^ We documented evidence of effect modification of fibre in the development of CD by smoking status, with inverse associations documented with higher intakes of cereal fibre in non-smokers and the odds of developing CD. The reasons for this association are unknown, and indeed may represent a false-positive result as multiple associations were analysed. Residual confounding may be another explanation, in that there is another exposure associated with cereal fibre intake which explains the inverse association. However, this result may be a real one as the inverse effect sizes were large with a biological gradient derived from dietary data recalled before the diagnosis of CD. This finding may suggest cereal fibre is unable to prevent any pathogenic effects of smoking; although the mechanism for this is unknown, it may be related to smoking effects of nicotine and/or other products of combusted tobacco on oxidative stress and the vascular circulation.^28^

This study had several methodological strengths which give support to the validity of our findings. Principally, the prospective design with the recording of habitual diet and smoking status at study entry before diagnosis minimized recall bias of participants for their fibre intakes, a bias which occurs in case-control investigations. Selection bias was unlikely due to the follow-up design. To reduce measurement bias, all potential identified cases were confirmed by clinicians who reviewed the medical notes. Follow-up bias should be low, i.e. most participants developing IBD are identified, as the numbers of cases diagnosed were similar to that expected to have accrued in this cohort, based on previously reported incidence studies.^29^ A limitation is that the relatively low number of cases available for study may have resulted in firstly weaker associations with dietary fibre going undetected and secondly a lack of statistical precision, which may be particularly relevant for assessing if there is a true inverse association of fibres, particularly from cereals, in non-smokers who developed CD. Follow-up of the cohort is continuing to accrue more cases to detect any weaker associations and gain greater statistical precision. Measurement error in the FFQ for dietary intake may occur if participants change their diet over time, and we only had access to dietary questionnaires from participants at recruitment. However, other longitudinal studies that repeated dietary measures over time showed minimal temporal changes. In the Netherlands Cohort Study on diet and cancer, intakes were compared between FFQs completed at baseline and then 5 years later, and reported a 2% decline in fibre intake in men and 5% in women.^30^ Here, the time between repeating the dietary assessments was similar to the median intervals between recruitment into EPIC-IBD and the development of CD and UC, in cohorts of similar ages at recruitment. As our dietary analyses were performed according to quintiles, this minimum dietary change over time will mean most participants remain in the same category of dietary intake during follow-up. Furthermore, other environmental risk factors that may influence the risk of IBD, but were not collected in this study, such as breast-feeding and antibiotic use, could confound associations, particularly for cereal fibre intake in non-smokers and the development of CD. When considering the generalizability of our findings, the population studied comprised mainly middle-aged to elderly participants in whom environmental factors appear to play a lesser role than in early-onset IBD.^31^ Therefore, whether fibre intake is involved in the aetiology of IBD in younger people is uncertain. However, our findings may be applicable to IBD diagnosed in younger patients in that: both genders were studied, the sites of distribution of inflammation in the gastrointestinal tract were similar, and the intake of fibre, as measured by cereal intakes, the major source of fibre, remains relatively stable as people age.^32^ The cohort in the UK, administered from Oxford, is composed of members of vegetarian societies and readers of health food magazines, whose inclusion could affect the generalizability of our findings. However, the proportions of the number of all cases diagnosed in this sub-cohort were low at 3.8% for CD and 6.8% for UC, and the median intakes of fibre between these UK individuals and those in the rest of the cohort were similar. Removing these participants from the analyses did not significantly alter any of the effect sizes or statistical significance of any associations.

Only one other prospective cohort study, to the best of our knowledge, has investigated dietary fibre and the aetiology of IBD. Here, in the US Nurses’ Health Studies [NHS] dietary, data were collected from 170776 women, aged between 25 and 55 years at recruitment, who were followed up for 26 years.^12^ The dietary information was derived from a semi-quantitative food frequency questionnaire completed every 4 years. During the follow-up period, 269 incident cases of CD and 338 cases of UC were confirmed through review of medical records. As in the EPIC-IBD study, no associations were found with either total fibre intake or fibre from fruits, vegetables, cereals or legumes and the development of UC. However, for CD, intake of the highest quintile of dietary fibre was inversely associated with risk [HR = 0.59, 95% CI = 0.39–0.90], with the apparent reduction derived from fibre intake from fruits, but not other sources. Possible reasons for the differences in results for fibre and CD between the two cohort studies may be that the US NHS, being larger, includes only women, and if fibre deficiency is involved in the aetiology near the time symptoms develop, this may go uncaptured in some participants developing IBD in this investigation from Europe. In the EPIC-IBD study, diet was recorded only once at recruitment, at a median time of approximately 5 years prior to diagnosis but up to 15 years, whereas in the US study food frequency questionnaires were administered every 4 years. However, as discussed above, as dietary changes are probably small this may not explain the differences in the findings. Therefore, overall the evidence would suggest fibre is not involved in the development of UC, but we need clarification of whether higher intakes of fibre are involved in protecting against the development of CD, perhaps from certain sources and in groups.

In summary, this prospective EPIC-IBD cohort study found no clear associations with the intakes of total fibre, or that from specific sources, and the development of either CD or UC. For cereal fibre there was evidence of effect modification from smoking. Continued follow-up of this cohort is occurring to see if there are weaker associations for fibre and to gain greater statistical precision of several estimates for CD. The results from both the present and the NHS prospective studies suggest fibre from different foods should be measured in future aetiological epidemiological studies of CD. Consistent findings of inverse associations with particular types of fibre may suggest aetiological mechanisms for fibre in the aetiology of CD.

## Funding

This study was supported by The Sir Halley Stewart Trust, Crohn’s and Colitis UK and The NHS Executive Eastern Region. SSMC is supported by an NIHR clinical lectureship. VAN is supported by Knud and Edith Eriksens Mindefond. The coordination of EPIC is financially supported by the European Commission [DG-SANCO] and the International Agency for Research on Cancer. The national cohorts are supported by the Danish Cancer Society [Denmark]; German Cancer Aid, Federal Ministry of Education and Research [Germany]; Dutch Ministry of Health, Welfare and Sports, Dutch Prevention Funds, LK Research Funds, Dutch ZON [Zorg Onderzoek Nederland], World Cancer Research Fund [WCRF], Statistics Netherlands [the Netherlands]; Swedish Cancer Society, Swedish Scientific Council and Regional Government of Skane and Västerbotten [Sweden]; and Cancer Research UK, Medical Research Council [UK].

## Conflict of Interest

All the authors declare no conflicts of interests.

## Author Contributions

VAN, SSC and ARH designed the study and VAN wrote the first draft of the paper. SSC and ARH recruited the centres and analysed the data. RL generated the master dataset, performed data entry, provided support on statistical analysis and contributed to writing the paper. The remaining co-authors are principal investigators in their respective centres who contributed to the local design, development and recruitment of participants into their cohorts and case identification and verification. These authors generated the local IBD databases, and contributed to the analysis and writing of the manuscript. All authors approved the final version of the manuscript.

## Appendix 1.

**Table:**

Country and centre	Size of cohort	Nature of cohort	Number of cases of incident CD	Number of cases of incident UC
United Kingdom				
Norfolk	25639	Population-based cohort of men and women aged 45–74 years. Recruited between 1993 and 1997. Cases identified up to June 2004 from follow-up questionnaires, in-patient admission data and histopathology records.	11	22
Oxford	50070	Members of UK vegetarian societies and readers of health food magazines [78% women], aged 20–80 years recruited between 1994 and 1999. Cases identified up to May 2004 by follow-up questionnaires.	4	15
Germany				
Heidelberg	25540	Population-based cohort of men aged 45–65 years and women aged 35–65 years. Recruited between 1994 and 1998. Cases identified up to June 2007 from follow-up questionnaires.	9	4
Potsdam	27548	Population-based cohort, men and women, aged 35–64 years. Recruited between 1994 and 1998. Cases identified up to April 2007 from follow-up questionnaires.	4	13
Italy				
Florence	13583	Population-based cohort, men and women aged 34–64 years. Recruitment between 1993 and 1998. Cases identified from regional database of IBD up to May 2004.	2	8
Ragusa	6403	Population-based cohort, men and women aged 34–65 years recruited between 1993 and 1997. Cases identified up to end of 2010 from follow-up questionnaires, in-patient admission data and histopathology records.	3	16
Sweden				
Umeå	25732	Population-based cohort, men and women aged 30–60 years. Recruited between 1992 and 1996. Cases identified up to February 2007 from regional database of IBD.	9	14
Malmö	28098	Population-based cohort, men and women aged 45–69 years. Recruited between 1991 and 1996. Cases identified up to October 2003 from regional database ofIBD.	11	21
Denmark				
Aarhus and Copenhagen	57053	Population-based cohort of men and women aged 50–64 years. Recruited between 1993 and 1997. Cases identified up to July 2007 from national database of IBD.	11	40
France				
Regions throughout the country	72996	Women aged 40–65 years recruited between 1990 and 1993 who are members of a health insurance scheme for school teachers and co-workers. Cases identified up to July 2009 by follow-up questionnaires.	18	22
The Netherlands				
Amsterdam, Doetinchem, Maastricht and Utrecht	40092	Men and women, aged 20–70 years recruited between 1993 and 1997 from the general population of three cities [Amsterdam, Doetinchem, Masstricht] and also the breast cancer screening programme in Utrecht. Cases identified up to December 2009 by regional IBD databases.	17	40
Greece	28572	Population-based cohort of men and women aged 29–76 years recruited between 1994 and 1999 from 11 regions throughout Greece. Cases identified up to September 2011 from follow-up questionnaires and histopathology records.	5	6
Total	401326		104	221

CD, Crohn’s disease; UC, ulcerative colitis; IBD, inflammatory bowel disease.

## References

[1] Molodecky NA, Soon IS, Rabi DM Increasing incidence and prevalence of the inflammatory bowel diseases with time, based on systematic review. Gastroenterology 2012;142:46–54.e42; quiz e30. 22001864 10.1053/j.gastro.2011.10.001

[2] Andersen V, Ernst A, Christensen J The polymorphism rs3024505 proximal to IL-10 is associated with risk of ulcerative colitis and Crohns disease in a Danish case-control study. BMC Med Genet 2010;11:82. 20509889 10.1186/1471-2350-11-82 PMC2891714

[3] Molodecky NA, Panaccione R, Ghosh S, Barkema HW, Kaplan GG; Alberta Inflammatory Bowel Disease Consortium Challenges associated with identifying the environmental determinants of the inflammatory bowel diseases. Inflamm Bowel Dis 2011;17:1792–9. 21744435 10.1002/ibd.21511

[4] Hou JK, Abraham B, El-Serag H Dietary intake and risk of developing inflammatory bowel disease: a systematic review of the literature. Am J Gastroenterol 2011;106:563–73. 21468064 10.1038/ajg.2011.44

[5] Andersen V, Olsen A, Carbonnel F, Tjønneland A, Vogel U Diet and risk of inflammatory bowel disease. Dig Liver Dis 2012;44:185–94. 22055893 10.1016/j.dld.2011.10.001

[6] Andersen V, Hansen AK, Heitmann AB Potential impact of diet on treatment effect from anti-TNF drugs in inflammatory bowel disease. Nutrients 2017;9:E286 28294972 10.3390/nu9030286 PMC5372949

[7] Vinolo MA, Rodrigues HG, Nachbar RT, Curi R Regulation of inflammation by short chain fatty acids. Nutrients 2011;3:858–76. 22254083 10.3390/nu3100858 PMC3257741

[8] Hartog A, Belle FN, Bastiaans J A potential role for regulatory T-cells in the amelioration of DSS induced colitis by dietary non-digestible polysaccharides. J Nutr Biochem 2015;26:227–33. 25498760 10.1016/j.jnutbio.2014.10.011

[9] De Filippo C, Cavalieri D, Di Paola M Impact of diet in shaping gut microbiota revealed by a comparative study in children from Europe and rural Africa. Proc Natl Acad Sci U S A 2010;107:14691–6. 20679230 10.1073/pnas.1005963107 PMC2930426

[10] Nagalingam NA, Lynch SV Role of the microbiota in inflammatory bowel diseases. Inflamm Bowel Dis 2012;18:968–84. 21936031 10.1002/ibd.21866

[11] Roberts CL, Keita AV, Duncan SH Translocation of Crohn’s disease *Escherichia coli* across M-cells: contrasting effects of soluble plant fibres and emulsifiers. Gut 2010;59:1331–9. 20813719 10.1136/gut.2009.195370 PMC2976079

[12] Ananthakrishnan AN, Khalili H, Konijeti GG A prospective study of long-term intake of dietary fiber and risk of Crohn’s disease and ulcerative colitis. Gastroenterology 2013;145:970–7. 23912083 10.1053/j.gastro.2013.07.050 PMC3805714

[13] Spooren CE, Pierik MJ, Zeegers MP, Feskens EJ, Masclee AA, Jonkers DM Review article: The association of diet with onset and relapse in patients with inflammatory bowel disease. Aliment Pharmacol Ther 2013;38:1172–87. 24118051 10.1111/apt.12501

[14] Richman E, Rhodes JM Review article: Evidence-based dietary advice for patients with inflammatory bowel disease. Aliment Pharmacol Ther 2013;38:1156–71. 24102340 10.1111/apt.12500

[15] Mahid SS, Minor KS, Soto RE, Hornung CA, Galandiuk S Smoking and inflammatory bowel disease: a meta-analysis. Mayo Clin Proc 2006;81:1462–71. 17120402 10.4065/81.11.1462

[16] European Prospective Investigastion into Cancer and Nutrition EPIC 2014 http://epic.iarc.fr/ Accessed April 10, 2016.

[17] Riboli E Nutrition and cancer: background and rationale of the European Prospective Investigation into Cancer and Nutrition (EPIC). Ann Oncol 1992;3:783–91. 1286041 10.1093/oxfordjournals.annonc.a058097

[18] Riboli E, Hunt KJ, Slimani N European Prospective Investigation into Cancer and Nutrition (EPIC): study populations and data collection. Public Health Nutr 2002;5:1113–24. 12639222 10.1079/PHN2002394

[19] Opstelten JL, Leenders M, Dik VK Dairy products, dietary calcium, and risk of inflammatory bowel disease: results from a european prospective cohort investigation. Inflamm Bowel Dis 2016;22:1403–11. 27120568 10.1097/MIB.0000000000000798

[20] Hart AR, Luben R, Olsen A Diet in the aetiology of ulcerative colitis: a European prospective cohort study. Digestion 2008;77:57–64. 18349539 10.1159/000121412

[21] Chan SS, Luben R, Olsen A Association between high dietary intake of the *n*-3 polyunsaturated fatty acid docosahexaenoic acid and reduced risk of Crohn’s disease. Aliment Pharmacol Ther 2014;39 :834–42. 24611981 10.1111/apt.12670 PMC4114542

[22] Murphy N, Norat T, Ferrari P Dietary fibre intake and risks of cancers of the colon and rectum in the European prospective investigation into cancer and nutrition (EPIC). PLoS One 2012;7:e39361. 22761771 10.1371/journal.pone.0039361 PMC3382210

[23] Slimani N, Ferrari P, Ocké M Standardization of the 24-hour diet recall calibration method used in the european prospective investigation into cancer and nutrition (EPIC): general concepts and preliminary results. Eur J Clin Nutr 2000;54:900–17. 11114689 10.1038/sj.ejcn.1601107

[24] Bank S, Skytt Andersen P, Burisch J Polymorphisms in the inflammatory pathway genes TLR2, TLR4, TLR9, LY96, NFKBIA, NFKB1, TNFA, TNFRSF1A, IL6R, IL10, IL23R, PTPN22, and PPARG are associated with susceptibility of inflammatory bowel disease in a Danish cohort. PLoS One 2014;9:e98815. 24971461 10.1371/journal.pone.0098815 PMC4074037

[25] Andersen V, Holst R, Kopp TI, Tjønneland A, Vogel U Interactions between diet, lifestyle and IL10, IL1B, and PTGS2/COX-2 gene polymorphisms in relation to risk of colorectal cancer in a prospective Danish case-cohort study. PLoS One 2013;8:e78366. 24194923 10.1371/journal.pone.0078366 PMC3806836

[26] Andersen V, Egeberg R, Tjønneland A, Vogel U Interaction between interleukin-10 (IL-10) polymorphisms and dietary fibre in relation to risk of colorectal cancer in a Danish case-cohort study. BMC Cancer 2012;12:183. 22594912 10.1186/1471-2407-12-183 PMC3472168

[27] Costea I, Mack DR, Lemaitre RN Interactions between the dietary polyunsaturated fatty acid ratio and genetic factors determine susceptibility to pediatric Crohn’s disease. Gastroenterology 2014;146:929–31. 24406470 10.1053/j.gastro.2013.12.034

[28] Cosnes J Tobacco and IBD: relevance in the understanding of disease mechanisms and clinical practice. Best Pract Res Clin Gastroenterol 2004;18:481–96. 15157822 10.1016/j.bpg.2003.12.003

[29] Shivananda S, Lennard-Jones J, Logan R Incidence of inflammatory bowel disease across Europe: is there a difference between north and south? Results of the European Collaborative Study on Inflammatory Bowel Disease (EC-IBD). Gut 1996;39:690–7. 9014768 10.1136/gut.39.5.690 PMC1383393

[30] Goldbohm RA, van ‘t Veer P, van den Brandt PA Reproducibility of a food frequency questionnaire and stability of dietary habits determined from five annually repeated measurements. Eur J Clin Nutr 1995;49:420–9. 7656885

[31] Taleban S, Colombel JF, Mohler MJ, Fain MJ Inflammatory bowel disease and the elderly: a review. J Crohns Colitis 2015;9:507–15. 25870198 10.1093/ecco-jcc/jjv059

[32] Henderson L, Gregory J, Irving K The National Diet and Nutrition Survey: adults aged 19–64 years. Office for National Statistics, Food Standards Agency 2003;2:67–76.

